# Extracellular Vesicles Modulate Liver Cells Viability and Reactive Oxygen Species in Patients Following a Very Low-Calorie Ketogenic Diet

**DOI:** 10.3390/nu16152386

**Published:** 2024-07-23

**Authors:** Francesco Balestra, Roberto Negro, Maria De Luca, Nicoletta Depalo, Federica Rizzi, Giorgia Panzetta, Valentina Arrè, Rita Mastrogiacomo, Sergio Coletta, Dolores Stabile, Pasqua Letizia Pesole, Nicole Cerabino, Martina Di Chito, Endrit Shahini, Gianluigi Giannelli, Giovanni De Pergola, Maria Principia Scavo

**Affiliations:** 1 Laboratory of Molecular Medicine, National Institute of Gastroenterology IRCCS “S. de Bellis”, Research Hospital, Via Turi 27, 70013 Castellana Grotte, Italy; francesco.balestra@irccsdebellis.it (F.B.); maria.deluca@irccsdebellis.it (M.D.L.); giorgia.panzetta@irccsdebellis.it (G.P.); 2 Laboratory of Personalized Medicine, National Institute of Gastroenterology IRCCS “S. de Bellis”, Research Hospital, Via Turi 27, 70013 Castellana Grotte, Italy; roberto.negro@irccsdebellis.it (R.N.); valentina.arre@irccsdebellis.it (V.A.); 3 Institute for Chemical-Physical Processes, Italian National Research Council (IPCF)-CNR SS Bari, Via Orabona 4, 70125 Bari, Italy; n.depalo@ba.ipcf.cnr.it (N.D.); f.rizzi@ba.ipcf.cnr.it (F.R.); rita.mastrogiacomo@uniba.it (R.M.); 4 National Interuniversity Consortium of Materials Science and Technology (INSTM), Bari Research Unit, Via Orabona 4, 70125 Bari, Italy; 5 Department of Chemistry, University of Bari Aldo Moro, Via Orabona 4, 70125 Bari, Italy; 6 Department of Pathology, National Institute of Gastroenterology IRCCS “S. de Bellis”, Research Hospital, Via Turi 27, 70013 Castellana Grotte, Italy; sergio.coletta@irccsdebellis.it (S.C.); dolores.stabile@irccsdebellis.it (D.S.); letizia.pesole@irccsdebellis.it (P.L.P.); 7 Center of Nutrition for the Research and the Care of Obesity and Metabolic Diseases, National Institute of Gastroenterology IRCCS “S. de Bellis”, Via Turi 27, 70013 Castellana Grotte, Italy; nicole.cerabino@irccsdebellis.it (N.C.); martina.dichito@irccsdebellis.it (M.D.C.); giovanni.depergola@irccsdebellis.it (G.D.P.); 8 Gastroenterology Unit, National Institute of Gastroenterology IRCCS “S. de Bellis”, Research Hospital, Via Turi 27, 70013 Castellana Grotte, Italy; endrit.shahini@ircllicsdebes.it; 9 Scientific Direction, National Institute of Gastroenterology IRCCS “S. de Bellis”, Research Hospital, Via Turi 27, 70013 Castellana Grotte, Italy; gianluigi.giannelli@irccsdebellis.it

**Keywords:** VLCKD, liver fibrosis, extracellular vesicles, ROS, cell proliferation

## Abstract

The VLCKD is a diet recognized to promote rapid fat mobilization and reduce inflammation, hepatic steatosis, and liver fibrosis. Extracellular vesicles (EVs) mediate cell-to-cell communication. The aim of the study is to investigate the role of circulating EVs in cell proliferation, ketone bodies, and ROS production in patients on an 8-week VLCKD regimen. Participants were classified as responders (R) or non-responders (NR) to VLCKD treatment based on their fibroscan results. In vitro experiments with the hepatic cell lines HEPA-RG (normal hepatocytes) and LX-2 (stellate cells) were conducted to investigate the effects of circulating EVs on cell viability, ROS production, and ketone body presence. The findings reveal a notable reduction in cell viability in both cell lines when treated with exosomes (EXOs). In contrast, treatment with microvesicles (MVs) did not appear to affect cell viability, which remained unchanged. Additionally, the levels of ketone bodies measured in urine were not consistently correlated with the reduction of fibrosis in responders (R). Similarly, an increase in ketone bodies was observed in non-responders (NR), which was also not aligned with the expected reduction in fibrosis. This inconsistency stands in stark contrast to the levels of Reactive Oxygen Species (ROS), which exhibited a clear and consistent pattern in accordance with the dietary intervention. Finally, in this preliminary study, ROS has been identified as a potential diet adherence marker for VLCKD patients; the ROS levels reliably follow the progression of the fibrosis response, providing a more accurate reflection of the therapeutic effects.

## 1. Introduction

The earliest documented administration of the ketogenic diet (KD) dates back to 1931 [1], when it was employed in patients to mitigate post-surgical effects associated with the administration of anesthetics. Subsequently, the KD was used as an anticonvulsant treatment for children with refractory epilepsy [2]. This therapeutic effect is attributed to a metabolic shift in the brain from a glucose-based energy substrate to a ketone-based substrate. Nowadays, a Very Low-Calorie Ketogenic Diet (VLCKD) is used in clinical procedures in patients that need to lose weight; it is characterized by a low carbohydrate content (<50 g/day), 1–1.5 g of protein/kg of ideal body weight, 15–30 g of fat/day, and a daily intake of about 500–800 calories [3]. In addition, it is associated with favorable biomarker and instrumental analysis changes, such as a reduction of liver fibrosis detected with a fibroscan [4] and a reduction of the inflammatory process [5]. Liver degeneration is among the earliest evidences in overweight and obese patients, whose causes are to be found in the crosstalk between all the cells that make up the liver tissue [6]. The critical event in the progression of liver disease is hepatocyte cell death, due to inflammation leading to fibrosis. The main cells involved in these two processes are hepatocytes and stellate cells [7]. Damaged Hepatocytes engage in endocrine and paracrine actions by producing extracellular vesicles (EVs) that carry pro-inflammatory cytokines, such as Transforming Growth Factor β1 (TGF-β1). TGF-β1 is a key profibrogenic cytokine and extracellular matrix component. In particular, this cytokine offers a promising target for the treatment of fibrosis [8], and it affects stellate cells, activating them to produce extracellular matrix components. This results in increased fibrosis, characterized by enhanced collagen synthesis and deposition [9]. Furthermore, TGF-β1 is clearly involved in the suppression of hepatocyte proliferation and has been shown to be produced by a multitude of non-parenchymal liver cells [9,10]. An important mechanism that involves TGF-β1 is reciprocal regulation with Reactive Oxygen Species (ROS), which has a central role during fibrotic degeneration of liver tissue [11]. Furthermore, TGF-β1 increases ROS production by inducing NADPH oxidases in the mitochondria, causing a redox imbalance by suppressing the antioxidant system. On the other hand, several studies have shown that ROS upregulates TGF-β1 gene expression [12]. Several studies have reported that connective tissue growth factor (CTGF) regulates ECM [13] production when cells are treated with TGF-β1 [14]. CTGF is also involved in various cellular processes, including proliferation, differentiation, migration, adhesion, and interactions with matrix components [15,16]. It is important to consider the role of extracellular vesicles (EVs), including exosomes (EXOs) and microvesicles (MVs), in intercellular communication within the liver [13]. Their diameter ranges from 30 to 1000 nm, and after release into the extracellular space, they are capable of transferring information from cells to other cells. Furthermore, the EVs are involved in pathological conditions, particularly several inflammatory diseases, and can cause injury to hepatocytes due to their pro-inflammatory cargo [14]. This cargo includes an imbalance between fatty acids, such as palmitic acid (pro-fibrotic) and oleic acid (anti-fibrotic), which can result from improper diets and excessive calorie intake [15]. We have demonstrated that treating overweight or obese subjects with a VLCKD leads to a reduction in white blood cells (WBCs) and platelets, along with decreasing inflammation, liver steatosis, and fibrosis [16]. There is no in vivo evidence regarding the role of the extracellular vesicles (EVs) and their cargo on the liver when patients follow a VLCKD. The aim of the study is to investigate the effect of EVs on hepatocytes and stellate cells (HEPA-RG and Lx-2 models) viability as well as ROS production in patients following a VLCKD for 8 weeks.

## 2. Materials and Methods

### 2.1. Patients and Study Design

The 8-week study was conducted by our Center of Nutrition for the Research and Care of Obesity and Metabolic Diseases of the National Institute of Gastroenterology at Saverio De Bellis Research Hospital (Castellana Grotte, Bari, Apulia, Italy). Seventy-eight patients were enrolled in this study. Inclusion criteria were age between 18 and 64 years, randomized in females (F) and males (M), body mass index (BMI) ≥ 25 kg/m^2^, and no current integrative or pharmaceutical treatment. The exclusion criteria included a list of contraindications for a Very Low-Calorie Ketogenic Diet (VLCKD): hypersensitivity to components contained in meal replacement products, type 1 diabetes mellitus, a history of cerebrovascular and cardiac diseases, respiratory insufficiency, severe GI diseases (e.g., inflammatory bowel disease, autoimmune diseases, cancer), chronic kidney disease characterized by an estimated glomerular filtration rate < 60, psychiatric issues, or pregnancy and lactation. In addition to the above-mentioned contraindications, other exclusion criteria included eating disorders and other serious mental illnesses, liver failure, substance abuse, frail elderly patients, active/severe infections, and rare disorders such as porphyria or deficiencies of carnitine, carnitine–palmitoyl transferase, or carnitine–acylcarnitine translocase or pyruvate carboxylase, and disorders of mitochondrial fatty acid oxidation. Patients who consumed more alcohol than recommended were also excluded. The assessment of daily alcohol consumption was conducted through direct questioning during the medical history assessment and was defined as follows: The question “Do you drink more than two glasses of alcohol per day?” was asked of male patients, and “Do you drink more than one glass of alcohol per day?” to female patients, in accordance with American and European recommendations regarding the daily consumption of alcohol. These recommendations indicate a recommended daily alcohol intake of 20 g for women and 30 g for men. The subjects were also questioned about smoking. This study was approved by the local Medical Ethics Committee (Prot. n. 170/CE De Bellis) and was conducted in compliance with the 1964 Helsinki Declaration. Written consent was collected prior to participating in the study. NCT05477212 is the study’s ClinicalTrials.gov identifier. Overweight or obese patients were enrolled from July 2022 to December 2023, and two check-ups were conducted, the first (T0) before starting with the VLCKD diet treatment and the second after eight weeks following the VLCKD treatment (T1). The VLCKD protocol was the one performed by Rinaldi et al., as previously described [17]. All patients followed the VLCKD (650–800 kcal/day), low-fat (only 20 g per day), and low-carbohydrate (<30 g per day from vegetables) diet, basing the initial phase of the diet only on olive oil. To verify their adherence to the diet, we asked the patients to fill out the dietary diary. In the first diet phase, patients were advised to drink two liters of water per day, and since the diet is unbalanced in terms of several dietetic components, micronutrients were supplied during the duration of the dietary regimen. The permitted foods were vegetables with a low glycemic index and low sugar content with the recommended amounts of fiber, herbs and spices, extra virgin olive oil (two tablespoons per day), and lemon flavoring. During phase 2, a small increase in calorie consumption was allowed. Blood samples were collected between 8:00 and 9:00, and biochemical evaluation of the concentrations of AST, ALT, and gamma-GT [18] was performed. Urine samples were also collected at T0 and T1 from all patients to analyze the presence of ketone bodies due to the diet. Briefly, a drop of fresh urine sample was deposited on the strip based on Ketone Sodium Nitroprusside 7.1% *w*/*w* to reveal ketone bodies (Just fitter Ketone Test strips, Chicago, IL, USA). After 15 s, excess liquid was shaken off, and the resulting color of the strip was compared with the color chart. Body mass index (BMI) (kg/m^2^) was measured for every patient using the same calibrated scale and stadiometer. Every anthropometric measure was taken at baseline and eight weeks after the VLCKD treatment. The Bioelectrical Impedance Analysis (BIA) was done by a nutritionist in our institute using a BIA-101 analyzer, a single-frequency bioimpedance analyzer (50 kHz frequency; Akern Bioresearch, Florence, Italy), in accordance with the procedures set by the European Society of Parenteral and Enteral Nutrition (ESPEN) [19]. Briefly, the nutritionist instructed patients to abstain from eating and drinking for 12 h before the exam, and to refrain from exercising for 24 h. Fibrosis and steatosis stages were diagnosed using transient elastography (Fibroscan^®^, Echosens, Paris, France), a cost-effective ultrasound-based technique capable of accurately quantifying and staging hepatic steatosis and fibrosis across many individuals. Assessments were conducted after a minimum fasting period of 6 h. Participants were positioned supine with their right arm raised above their heads, and measurements were taken from the right liver lobe in the intercostal position. This method is straightforward and does not require extensive knowledge of B-mode ultrasonography. The FibroScan device measures liver elasticity (stiffness, kPa) using M and XL probes and steatosis through ultrasonic echo wave attenuation (CAP, db/m). Liver steatosis was confirmed when the CAP exceeded 275 db/m at the standard frequency of 3.5 MHz, and liver fibrosis was suspected when the stiffness value exceeded 7 kPA [20]. For liver stiffness, we used the interquartile range (IQR) as a percentage of the median (IQR/median%) to estimate variance, considering the results reliable if IQR/median% was less than 30%. A median of at least ten valid measurements, along with variance measurements, were obtained [21].

### 2.2. Extracellular Vesicles Extraction and Characterization

The EVs were extracted from patient sera using the modified previous setting protocol to obtain EXO and MVs [22]. Briefly, the serum was centrifuged at 1800× *g* for 10 min at 4 °C, and the supernatant was transferred into a new tube and centrifuged again at 3800× *g* for 15 min at 4 °C; the supernatant was transferred into a new tube for another centrifugation cycle at 12,500× *g* for 15 min at 4 °C. The pellet contained the MVs that were suspended in 100 μL of ultrapure water. Subsequently, an ultracentrifugation (UC) cycle was performed on the supernatant using a BECKMAN, L-60 Ultracentrifuge, at 75,000× *g* for 1 h at 4 °C; then the supernatant was transferred into another ultracentrifuge tube, and a second ultracentrifugation cycle was performed at 100,000× *g* for 1 h and 30 min. The pellet containing EXOs was collected and diluted in 200 μL of ultrapure water. After their extraction, the freshly obtained EXOs and MVs were characterized by Dynamic Light Scattering (DLS) analysis, ζ-Potential measurements, and (TEM). The other parts were then stored at −80 °C until the protein analysis was carried out. The TEM used to characterize EXOs and MVs was Jeol Jem-1011 (JEOL USA, Inc., Peabody, MA, USA), a transmission electron microscope working at an accelerating voltage of 100 kV, and the Olympus Quemesa Camera (11 Mpx) (Olympus, Shinjuku-ku, Tokyo 163-0914, Japan) to acquire proper images. A Zetasizer Nano ZS, Malvern Instruments Ltd., Worcestershire, UK (DTS 5.00), was employed to evaluate the hydrodynamic diameter, size distribution, and colloidal stability of EVs. All reported data are presented as mean values ± standard deviation (three replicates). The expression levels of proteins involved in inflammation and fibrosis, specifically TGF-β1 and CTGF, which play roles in the induction and progression of liver fibrosis and regulation of signal transduction, were analyzed using Western blotting. Protein concentrations were measured using a standard Bradford assay (Bio-Rad, Milan, Italy). Aliquots containing 20 µg of total protein extracts were loaded onto 4–15% precast polyacrylamide gels (Bio-Rad Laboratories, Milan, Italy). After electrophoresis, the proteins were transferred onto a polyvinylidene fluoride (PVDF) membrane (Bio-Rad Laboratories, Milan, Italy). The membranes were then probed with the following primary antibodies: anti-TGFβ1 (1:1000, Santa Cruz, CA, USA), anti-CTGF (1:500, Cell Signaling Technology, Beverly, MA, USA), anti-Annexin 1, anti-CD63, and anti-CD81 (all 1:1000, Cell Signaling Technology, Beverly, MA, USA). Following an overnight incubation, the membranes were washed and incubated with the appropriate secondary antibodies. Protein detection was carried out using enhanced chemiluminescence (ECL, Thermo Fisher Scientific, Waltham, MA, USA). The protein signals were visualized using the Chemidoc Molecular Imager (Bio-Rad, Milan, Italy) and normalized against, respectively, Annexin 1 and CD81 expression. Image analysis was performed using Image Lab 5.2.1 software. Analyses were conducted on samples extracted from EVs isolated from patients at T0 and T1.

### 2.3. Cell Culture

The HEPA-RG human hepatoma cell line (Thermo Fisher Scientific, Waltham, MA, USA) was cultured using a hepatocyte bullet kit medium supplemented with 10% FBS (exosome-depleted) from Lonza Biowhittaker, Oslo, Norway, and 1% Antibiotic-Antimycotic (penicillin 10,000 U/mL, streptomycin 10,000 U/mL) from the same supplier. The LX2 Human Hepatic Stellate cell line (Millipore, Merck Life Science, Milan, Italy) was cultured in Dulbecco’s Modified Eagle Medium (DMEM) (Thermo Fisher Scientific, Waltham, MA, USA) with 10% FBS (exosome-depleted), 2.5% HEPES 1M (*N*-2-hydroxyethylpiperazine-*N*-2-ethane sulfonic acid) (Gibco™, Thermo Fisher Scientific, Waltham, MA, USA), 1% Sodium Pyruvate 100 mM (Gibco™, Thermo Fisher Scientific, Waltham, MA, USA), and 1% Antibiotic-Antimycotic. For treatments with exosomes (EXOs) or microvesicles (MVs), both cell lines were cultured to form semi-confluent monolayers and treated with EXOs or MVs derived from patients. The treatment concentration was 20 μg/μL of extracellular vesicle (EV) proteins for 8, 24, 48, or 72 h, depending on the specific experimental requirements for transcriptomic or protein functionality analyses.

### 2.4. Cell Viability Assay

The hepatic cell lines LX-2 and HEPA-RG were seeded into 96-well plates at a density of 2 × 10^3^ cells per well. After 24 h, they were treated with extracellular vesicles (EVs) from the sera of responders (R) and non-responders (NR) at T0 and T1, incubated at 37 °C for 24, 48, 72, and 96 h. Untreated cells served as controls. Following EV treatment, both cell lines received 20 μL of MTS tetrazolium compound reagent (Promega, WI, USA) in a total volume of 120 μL and were incubated at 37 °C for 3 h. Absorbance was measured at 490 nm using a PerkinElmer Victor Plate Reader (Lier, Belgium). Cell viability percentage was calculated using the following equation:

% Viability = Mean OD samples/Mean OD CTR × 100.

### 2.5. Reactive Oxygen Species Detection

The HEPA-RG and LX-2 cell lines were analyzed for Reactive Oxygen Species (ROS) production using CellROX™ Orange reagent (Thermo Fisher Scientific) following the manufacturer’s instructions. CellROX Orange is a fluorogenic, cell-permeant probe that is non-fluorescent in its reduced state but exhibits bright orange fluorescence upon oxidation by ROS. This fluorescence is detectable with fluorescence microscopy at absorption/emission maxima of 545/565 nm.

Briefly, both cell lines were seeded in 96-well plates at a density of 1 × 10^3^ cells/well and cultured for 3 days at 37 °C. The cells were then treated for 48 h with EXOs or MVs derived from responders (R) and non-responders (NR), as described below. Both groups were further divided based on ketone body production status (positive (+) or negative (–)) before (T0) and after (T1) Very Low-Calorie Ketogenic Diet (VLCKD) administration.

The cells were subsequently incubated at 37 °C for 30 min in 5 μM CellROX Orange reagent, followed by three washes with DPBS. All steps were performed in the dark. The 96-well plates were examined using a Nikon Eclipse Ti2 fluorescence microscope, and images were captured using Kr-Ar and Ar lasers fitted with 555 nm (red channel) band-pass filters at 40× magnification. Image analysis was conducted with ImageJ software.

### 2.6. Statistical Analysis

ANOVA was performed to test for differences. In all analyses, control subjects at baseline served as the reference category. Statistical significance was defined as * *p* < 0.01 and **** *p* < 0.0001. Sigma Stat software was used for post-estimation analysis, employing the Bonferroni test for multiple comparisons.

## 3. Results

### 3.1. Patients Clinical Parameters after VLCKD

The classification of patients was conducted considering their responsiveness to the VLCKD, principally on the basis of fibrosis determination using the Fibro-Scan parameter measured in kilopascal (kPa) based on the values reported in Table 1, before the diet (T0) and after 8 weeks of VLCKD (T1).

**Figure 1:**
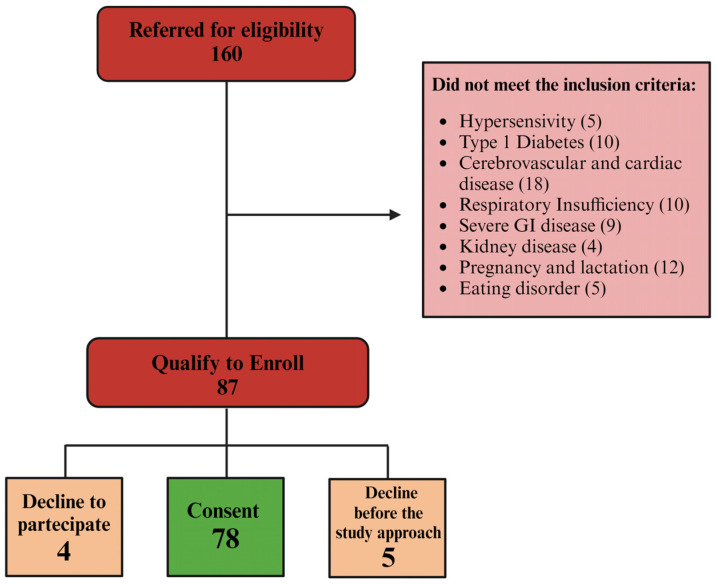
Flow diagram of patients’ enrollment. Out of 160 eligible patients, 73 did not meet the inclusion criteria, leaving 87 qualified candidates. Among these, four declined to participate, and five opted out at the start of the treatment. Ultimately, 78 patients were enrolled and completed the treatment.

Firstly, the physician verified the dietary diary of each patient to confirm their diet adherence. All patients were administered the International Physical Activity Questionnaire (IPAQ) to assess their daily energy expenditure. Patients were asked to maintain their usual physical activity levels throughout the study period. At the end of eight weeks, the IPAQ questionnaire was repeated, and it confirmed that the usual level of physical activity did not change. Patients were enrolled following the criteria of inclusion and exclusion and according to their will, as shown schematically in Figure 1.

**Figure 2:**
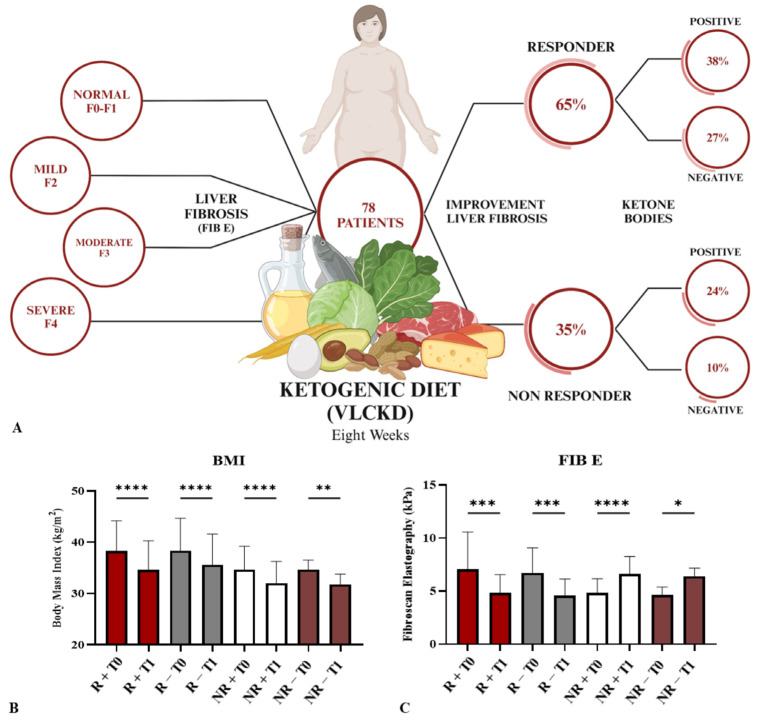
Patients’ characteristics. Stratification of patients for liver fibrosis considering FIB E (fibro scan elastography), the improvement of liver fibrosis, and ketone body production (**A**). Modification of BMI (Body Mass Index) in all groups of patients before and after diet, both R and NR patients (**B**). Schematic modification of FIB E in R and NR patients (**C**). * *p* < 0.01, ** *p* < 0.001, *** *p* < 0.0001, and **** *p* < 0.00001.

As shown in the scheme reported in Figure 2A, 51 patients (64.4% of enrolled patients) were classified as responders (R) due to the reduction of the fibrosis score after 8 weeks of diet, while 27 patients (34.6% of enrolled patients) were classified as non-responders (NR) due to an increase in fibrosis. All values reported in Table 2 are significant for a reduction in the group of R patients and for an increase in the group of NR patients (*p* < 0.05) of Fibroscan elastography (FIB E) (kPa). The average (Avg) value for all groups of patients is considered.

The parameters considered in each patient’s evaluation for this study are reported in Table 3. 

After classifying patients as R and NR, we evaluated the presence of ketone bodies in the urine derived from all patients. Ketone bodies were not present in all R patients. In particular, 30 R patients (38.46% of the total enrolled patients) were positive for ketone production and 21 were not (26.92% of the total enrolled patients), although all had a reduced fibrosis score and weight loss. In the same way as in R patients, NR patients underwent an additional stratification based on the production of ketone bodies. This analysis revealed that nineteen NR patients (24.36% of total enrolled patients) produced ketones, while the remaining eight NR patients (10.26% of total enrolled patients) showed no reduction in the fibrotic score and did not produce ketone bodies. All patients admitted to the dietary protocol lost weight, and so the BMI also declined. In particular, the R patients with a higher level of ketones in the urine showed a significant reduction in BMI (*p* < 0.0001), similar to the NR patients with positive ketone bodies (*p* < 0.0001). However, NR patients who did not present ketones in the urine also experienced significant weight loss and a corresponding BMI decline, but to a lesser extent than the other groups (*p* < 0.001) (Figure 2B). In addition to weight loss, in all R patients there was no evidence of ketone body production, which involves a significant reduction of FIB E (*p* < 0.0001), while NR patients showed a higher and more significant increase of FIB E in the presence of ketone bodies (Figure 2C) (*p* < 0.00001); when there were no ketone bodies in the urine, the increase of FIB E was less marked but still significant (*p* < 0.05).

### 3.2. Extracellular Vesicles Extraction and Characterization

**Figure 3:**
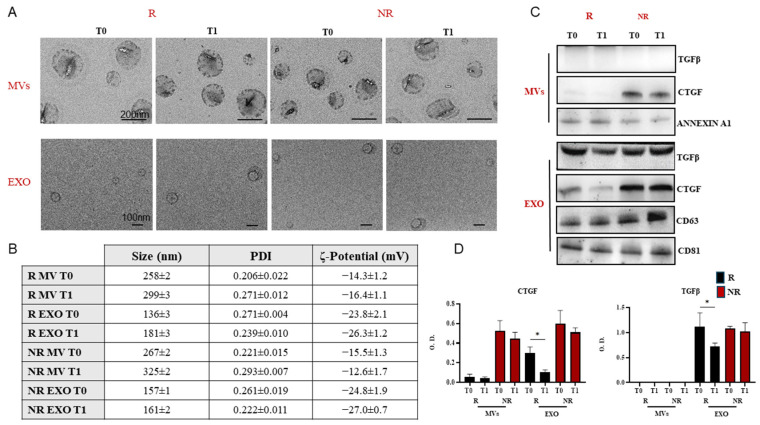
Characterization of EVs (extracellular vesicles) derived from VLCKD-treated patients. (**A**) TEM (Transmission Electron Microscopy) micrographs and (**B**) average hydrodynamic diameter and PDI (polydispersity index) obtained by DLS (Dynamic Light Scattering) analysis and ζ-potential value (mean ± SD) of EVs, both EXO (exosomes) and MVs (microvesicles), extracted from sera of VLCKD-treated patients, responders (R) and non-responders (NR), before (T0) and after (T1) VLCKD administration. Representative Western blotting of different proteins (TGF-β1, and CTGF), housekeeping proteins (Annexin A1 and CD81), and CD63 (**C**), in EXOs and MVs derived from patients treated with VLCKD. Semiquantitative evaluation of the considered protein expression levels in EXOs and MVs obtained from both R and NR by video-densitometry analysis of TGF-β1 and CTGF bands on Western blotting (**D**). The Annexin A1 and CD81 protein bands were used for normalization of the protein bands for each subject. (*) *p* < 0.01, while CD63 was used as CD81 to recognize the EXOs fraction.

TEM, DLS analysis, and ζ-Potential measurements were performed to characterize two subpopulations of freshly extracted EVs, namely EXOs and MVs, from the sera of patients following the VLCKD. These analyses were conducted before (T0) and after (T1) VLCKD administration to both R and NR patients to assess EV size, size distribution, morphology, and surface charge (Figure 3). TEM investigation revealed that the vesicles were spherical and nanosized, with a homogeneous size distribution of approximately 100 nm or less for EXOs and a polydisperse size distribution ranging from 150 nm to 400 nm for MVs (Figure 3A). DLS analysis showed that the average hydrodynamic diameter was greater than 250 nm for the MVs and less than 200 nm for the EXOs (Figure 3B). ζ-Potential measurements indicated that all EVs isolated, including both EXOs and MVs, were negatively charged due to the presence of negatively charged phospholipids on the EV membrane. DLS analysis revealed average hydrodynamic diameter values greater than 250 nm for the larger subpopulation of EVs (MVs) and less than 200 nm for the smaller subpopulation (EXO) (Figure 3B).

The presence of TGF-β1, a pleiotropic cytokine involved in both suppressive and inflammatory pathways, was investigated in EVs derived from patients with the highest fibrotic scores (F3–F4 for responders [R] and F2 for non-responders [NR]). Additionally, the evaluation included the presence of CTGF, a protein associated with extracellular matrix production, as shown in Figure 3C. As observed, MVs from both R and NR patients did not contain TGF-β1. Furthermore, CTGF was completely absent in the MVs derived from R patients. Although CTGF was present in the MVs from NR patients, there was no variation between the pre-diet (T0) and post-diet (T1) conditions. In contrast, EXOs displayed a significant reduction in TGF-β1 expression in R patients after the VLCKD regimen (T1), as evidenced by immunoblotting, whereas its expression levels in NR patients remained constant at T1 (Figure 3D) (*p* < 0.01). Concomitantly, in these R patients, a significant decrease in CTGF content was observed from T0 to T1 stage.

### 3.3. Effect of Exosomes on Liver Cell Viability

**Figure 4:**
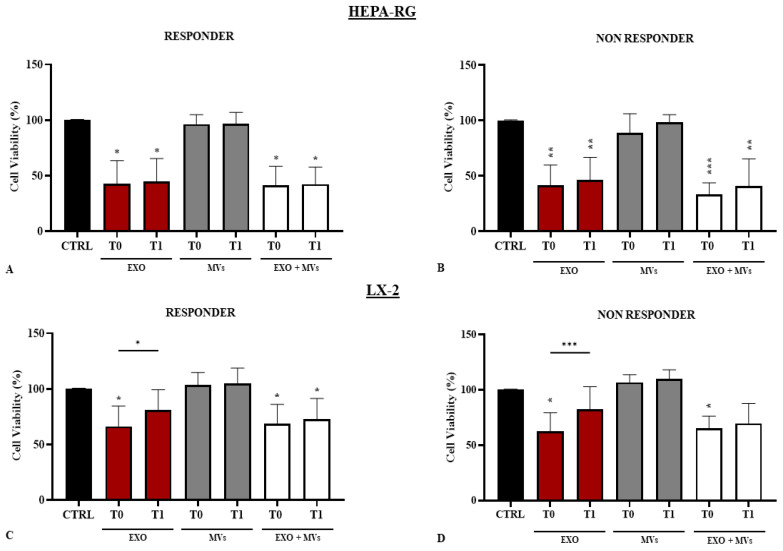
Cell viability in cells treated with EVs from R and NR patients. Cell viability was evaluated by MTS (3-(4,5-dimethylthiazol-2-yl)-5-(3-carboxymethoxyphenyl)-2-(4-sulfophenyl)-2H-tetrazolium) assay on Hepa-RG and LX-2 cells, respectively, after incubation with EXOs (exosomes) and MVs (microvesicles) derived from R (**A**,**C**) and NR (**B**,**D**) patients. The EXOs and MVs concentrations in terms of the total protein content were fixed at 20 µg/µL. Negative controls were untreated cells (CTR). Experiments were repeated three times. * *p* < 0.01, ** *p* < 0.001, and *** *p* < 0.0001.

HEPA-RG and LX-2 cell lines were treated with T0 and T1 EVs, both MVs and EXO isolated from R and NR patients after 96 h, and cell viability was determined using the MTS assay. HEPA-RG cell viability was significantly reduced (*p* < 0.01) following treatment with EXOs, with the combination of EXOs and MVs, but not with MVs used alone. This result was obtained using EXOs and MVs isolated from biological samples of R and NR patients, collected before (T0) and after (T1) the VLCKD diet. This suggests that the observed effects are due to EXOs but not MVs (Figure 4A,B).

The trend observed with the treatment of the LX2 cell line with the EVs from R is the same as that observed using HEPA-RG. It was noted that when cells are treated with the EXOs of R patients, the viability is lower compared with control (CTR) cells treated with EXOs extracted from patients before dietary treatment (T0) (*p* < 0.01). On the contrary, the cells treated with EXOs derived from R patients after VLCKD (T1) showed increased cell viability, which was significant compared with T0 (*p* < 0.01). When treated with MVs, the LX2 cells showed the same viability as the CTR. When the cells were treated with a combination of EXOs and MVs, the trend was like that observed with treatment using only EXOs. The reduction in cell viability was consistent with that seen in CTR cells (*p* < 0.01). These findings suggested that EXOs play a fundamental role in the toxic effect on cells (Figure 4C,D). As regards HEPA-RG, treatment with EXOs from NR patients at T0 induced a significant reduction in cell viability (*p* < 0.01), also in comparison with the CTR. Similarly, no effect on cell viability was observed after cell administration of R-derived or NR-derived MVs compared with the control cells. However, a significant reduction in viability was observed when using the combination of both EXOs and MVs derived from NR patients at T0 (*p* < 0.01). These results confirmed the toxic effect of TGF-β1 delivered in EXOs on HEPA-RG, which was less pronounced in the treatment of the LX2 cell line.

### 3.4. Modulation of Reactive Oxygen Species (ROS) in HEPA-RG and LX2 Treated with EXO and MVs

**Figure 5:**
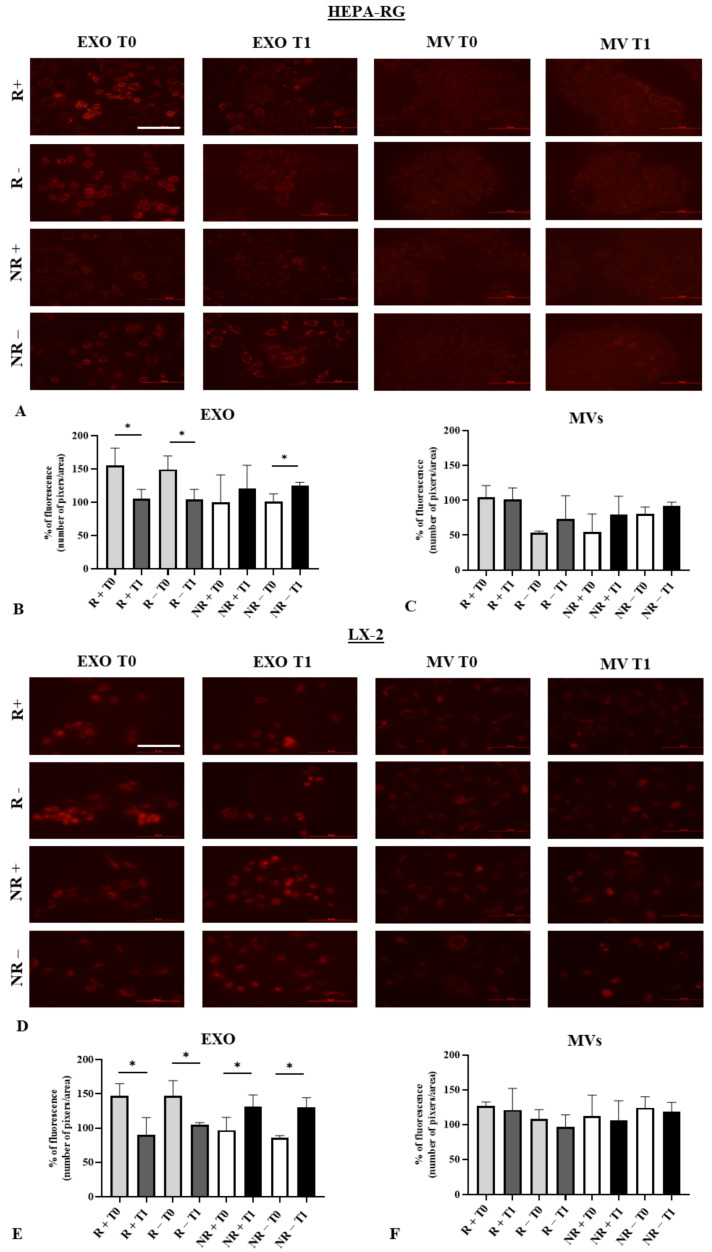
EVs (extracellular vesicles) treatment and ROS (Reactive Oxygen Species) variation in HEPA-RG and LX2. Representative fluorescent images taken by a fluorescence microscope of HEPA-RG (**A**) and LX2 (**D**). Intensity quantification of fluorescence in HEPA-RG (**B**,**C**) and LX2 (**E**,**F**) after treatment for 48 h with EXOs (exosomes) and MVs (microvesicles) derived from R and NR patients. Scale bar = 100 μm. * *p* < 0.01.

The ROS evaluation was conducted using the HEPA-RG and LX2 cell lines treated with EXOs and MVs derived from responder (R) and non-responder (NR) patients. Figure 5A shows representative fluorescence images of HEPA-RG cells treated with EXOs and MVs from R and NR patients before (T0) and after (T1) VLCKD administration. To compare ROS levels with ketone body presence, patients were stratified based on urine ketone bodies and further divided into R+ and R− (positive or negative for ketone bodies), as well as NR+ and NR−. A significant decrease in ROS production was observed in HEPA-RG cells treated with EXOs from both R+ and R− samples (Figure 5B, *p* < 0.01), whereas an increased trend was seen in cells treated with EXOs from NR patients. Specifically, EXOs from NR− patients showed a significant increase in ROS production (*p* < 0.01), independent of ketone body presence but proportional to TGF-β1 levels in EXOs from R and NR patients at T0 and T1. No ROS variation was observed in cells treated with MVs under any conditions (Figure 5C). The same experiment was conducted with LX2 cell lines, as shown in Figure 5D–F. A significant reduction in ROS between T0 and T1 was observed in LX2 cells treated with EXOs from R patients, regardless of ketone body presence (Figure 5D, *p* < 0.01). Conversely, a significant increase in ROS between T0 and T1 was found in LX2 cells treated with EXOs from NR patients, irrespective of ketosis (Figure 5E, *p* < 0.01). ROS levels in cells treated with MVs did not differ between conditions (Figure 5F). Overall, a reduction in ROS was observed in cells treated with EXOs from R patients, while an increase was noted in cells treated with EXOs from NR patients. These results were consistent with the patients’ responsiveness to the diets, analysed in relation to ketone body production.

## 4. Discussion

The ketogenic diet (KD) is based on a protocol characterized by high fat and protein intake with low carbohydrate consumption. Four types of ketogenic diets are used in clinical treatment: Classical KD typically consists of 90% fat, 4% carbohydrate, and 6% protein. Medium-chain triglyceride KD comprises 10% long-chain triglycerides fat, 60% medium-chain triglycerides fat, 20% carbohydrate, and 10% protein. Modified Atkins KD contains 65% fat, 10% carbohydrate, and 25% protein, and a low-glycemic index diet includes 60% fat, 10% carbohydrate, and 30% protein [23].

Variations of the classical KD include the Low-Calorie KD (LCKD) and the Very-Low-Calorie KD (VLCKD). The LCKD involves a calorie intake of 800 to 1200 kcal/day with 58% fat, 13% carbohydrate, and 29% protein. The VLCKD involves a caloric intake of under 800 kcal/day with less than 50 g/day of carbohydrate (13%), 1 to 1.5 g of protein per kg of body weight (44%), and 43% fat, which appear reduced compared with the KD [24]. These variations further reduce carbohydrate intake, prompting the body to switch to fatty acid oxidation and induce ketogenesis. Research indicates that the VLCKD improves heart failure [25], offers neuroprotective benefits for conditions such as schizophrenia, multiple sclerosis, Parkinson’s, and Alzheimer’s diseases [26,27], reduces inflammation [28], and helps in muscle force recovery post-critical illness [29,30].

Recently, the KD has gained popularity as an effective weight-loss method by reducing appetite through the production of ketone bodies [31]. However, unlike the VLCKD, it can, in some cases, dramatically increase LDL levels in patients [32,33]. Furthermore, several studies confirm the safety of the VLCKD, showing it significantly reduces LDL cholesterol levels, glucose, and triglycerides, as it happens in our patients even in patients with type 2 diabetes, and as demonstrated in this study after 8 weeks of treatment [16,34,35]. VLCKDs are diets with a higher level of calories from fat (± 90%) and a lower proportion from carbohydrates and proteins (±10%) [36]. This type of dietary approach is a non-pharmacological method currently used for chronic diseases such as Metabolic Dysfunction-Associated Steatotic Liver Disease (MASLD), several types of cancer, coronary disease, hypertension, metabolic diseases, and related co-morbidities like type 2 diabetes, overweight, and obesity [37]. In addition, ketosis produced by proven ketogenic diets also leads to an improvement in the symptoms of epileptic patients, probably because, in the switch from glucose to ketone, the body metabolism influences the regulation of certain neurotransmitters and oxidative stress [38,39]. For the first time, this study analyzes the role of EVs in the transportation of certain pro-inflammatory proteins, such as TGF-β1, and the fibrosis protein, CTGF. All these results are connected with the production and modulation of ROS when patients undergo a controlled diet based on the use of VLCKD. TGF-β1 is directly involved in apoptosis and the epithelial–mesenchymal transition (EMT) of hepatocytes [40]. Moreover, its role in fibrinogenesis is exerted through Smad pathways, which are involved in processes resulting in extracellular matrix (ECM) deposition and the release of regulatory proteins such as CTGF. CTGF, in turn, can regulate TGF-β1 production, creating a positive feedback loop. Both CTGF and its downstream signaling pathways are implicated in the development of liver inflammation and fibrosis, although the exact role of CTGF in diet-induced human liver fibrosis remains not entirely understood [41]. Additionally, it has also been reported in the literature that the ROS modulate TGF-β1 signals using the Smad pathways. At the same time, TGF-β1 increases ROS production and suppresses antioxidant enzymes, leading to a redox imbalance in a reciprocal regulation process [11,42]. These evidences confirm what we found experimentally using HEPA-RG and LX2 cells, treated with the EVs of patients, subdivided into R and NR to dietary therapy, as based on a decrease or increase of the FIB-E score. Indeed, in this study, we investigated the presence of TGF-β1 and CTGF in EVs derived from the plasma of patients following a VLCKD to provide insights, for the first time, on the role of TGF-β1 and CTGF-delivering EVs in the modulation of liver cell viability and ROS production in these patients. We demonstrated that the fraction of EVs responsible for the delivery of TGF-β1 and CTGF in all patients were EXOs, while MVs were not involved. The delivery of TGF-β1 by EXOs has been previously reported by G. V. Shelke et al., who demonstrated that EXOs released by mast cells harbor both active and latent TGF-β1 on their surfaces. This study showed that EXOs from mast cells can alter the phenotype and function of mesenchymal stem cells (MSCs) via TGF-β1 bound to the surface of the EXOs through proteoglycans [43].

Here, specifically, we observed that the presence of TGF-β1 and CTGF was reduced in EXOs derived from R patients compared with NR patients. In a previous study, our group showed a significant decrease in CAP, FIB-E, and BMI, resulting in a greater reduction in hepatic steatosis after VLCKD administration [17,44]. The VLCKD is well-known not only for weight loss but also for generating anti-inflammatory and anti-fibrotic ketone bodies [45,46]. These considerations highlight the potential use of novel prognostic biomarkers to monitor patients’ nutritional and therapeutic pathways, alongside the instrumental markers already in clinical use.

As previously described, the VLCKD induces the production of ketone body synthesis from acetyl-coenzyme A (acetyl-CoA), which is the first product of mitochondrial b-oxidation in the fatty liver [47]. Much caution must be employed when considering ketones as defined molecules to evaluate adherence to the diet of patients. The level of ketogenesis depends on the proportion of ketogenic to non-ketogenic nutrients consumed in the diet (e.g., several amino acids can be converted into glucose, making them glucogenic) [48]. Based on this concept, weight loss programs such as VLCKDs that have a low-fat, low-carb, and high protein makeup are not inherently ketogenic, but they can trigger ketosis through a substantial calorie reduction due to internal fat burning [49]. Considering this evidence, our finding that although all our patients followed the VLCKD, adhering to the diet regimen with weight loss and reduction of fibrosis, some of them had negative ketone production is not surprising.

Our results in the enrolled patients are in line with the literature, in which it is emphasized that the ketone bodies are not markers of adherence to the VLCKD diet, since in patients undergoing this type of diet regimen, there is not always an effective and detectable production of ketone bodies due to an increase in proteins with a rich glucogenic amino acid intake [50]. One of the negative factors related to the obese condition is an increase in oxidative stress due to mitochondrial damage by ROS [51]. 

## 5. Conclusions

In conclusion, on the basis of this study, we hypothesize that the use of ROS production may be a useful molecule for monitoring patients’ adherence to the diet without considering ketone body production. As observed in both cell lines overexpressing TGF-β1 receptors, when treated with EXOs and MVs from R and NR patients, both positive and negative for ketone production, we identified TGF-β1 or CTGF-delivering EXOs as the key agents. These EXOs were able to be internalized by the cells, presumably through fusion and receptor-mediated endocytosis. This uptake process facilitated modulation of ROS production based on their TGF-β1 and CTGF content, thereby exerting regulatory effects on fibrosis. Furthermore, in this preliminary study, the stratification of patients into R and NR groups based on the reduction of the fibrosis score after therapy and the VLCKD instead of the presence of ketone bodies allowed us to observe the potential of using ROS as a monitoring molecule to determine patient adherence to dietary treatment.

## Figures and Tables

**Table 1:** Fibrotic score. FibroScan elastography expressed in kPa to determine the fibrotic status, measuring liver stiffness (hardness) and fatty changes in liver. The different scores range from F0 to F4, from <7 to >14.

Fibroscan Elastography (kPa)	
F0–F1	<7
F2	7.1–10
F3	10.1–14
F4	>14

**Table 2:** Characteristics of patients. Subdivision of all patients into responders and non-responders on the basis of fibrotic score decrease. BMI = body mass index (kg/m^2^); FIB E (fibro scan elastography). M: male, F: female, Avg. M–F: Average between males and females.

	Sex	Weight (kg)			BMI (kg/m^2^)			FIB E (kPa)		
		T0	T1	*p*	T0	T1	*p*	T0	T1	*p*
**F0–F1**										
Responder	M	107.7 ± 14.0	98.8 ± 12.6	<0.0001	35.3 ± 4.0	32.5 ± 3.8	<0.0001	5.7 ± 1.0	3.9 ± 1.2	0.0008
	F	94.5 ± 12.1	86.1 ± 10.1	<0.0001	36.1 ± 3.8	33.0 ± 3.6	<0.0001	5.1 ± 1.0	4.0 ± 1.0	<0.0001
	Avg. M–F	98.5 ± 14.0	89.9 ± 12.8	<0.0001	36.0 ± 3.7	32.9 ± 3.6	<0.0001	5.2 ± 1.0	4.0 ± 1.0	<0.0001
Non-responder	M	96.6 ± 3.2	88.3 ± 2.9	0.0003	33.7 ± 1.7	31.0 ± 1.7	<0.0001	5.1 ± 0.9	5.6 ± 0.5	0.1532
	F	85.5 ± 5.0	78.8 ± 3.4	<0.0001	33.5 ± 2.4	30.9 ± 2.5	<0.0001	4.1 ± 0.8	5.7 ± 1.1	<0.0001
	Avg. M–F	88.3 ± 6.7	81.2 ± 5.4	<0.0001	31.6 ± 7.2	30.9 ± 2.2	<0.0001	4.4 ± 0.9	5.7 ± 1.0	<0.0001
**F2**										
Responder	M	132.2 ± 24.1	120.5 ± 23.8	0.0002	42.6 ± 8.0	39.0 ± 8.3	<0.0001	8.4 ± 0.9	6.1 ± 0.7	0.0015
	F	103.0 ± 12.8	94.8 ± 14.6	0.0874	39.0 ± 3.3	35.9 ± 4.4	0.0845	8.7 ± 0.9	6.0 ± 1.0	0.0456
	Avg. M–F	124.2 ± 25.0	113.5 ± 24.2	<0.0001	41.6 ± 7.1	38.1 ± 7.4	<0.0001	8.4 ± 0.7	6.0 ± 0.7	0.0002
Non-responder	M	86.8 ± 5.3	79.0 ± 5.0	0.0165	32.6 ± 0.2	29.6 ± 0.1	0.0212	6.1 ± 1.7	8.4 ± 1.8	0.0141
	F	99.4 ± 17.2	91.3 ± 15.0	0.0003	37.5 ± 5.2	34.4 ± 4.9	0.0002	5.3 ± 1.3	7.7 ± 0.8	0.0002
	Avg. M–F	97.1 ± 16.3	89.1 ± 14.4	<0.0001	36.6 ± 5.1	33.5 ± 4.8	<0.0001	5.4 ± 1.3	7.8 ± 0.9	<0.0001
**F3–F4**										
Responder	M	152.2 ± 22.8	141.2 ± 25.3	0.0187	48.5 ± 4.1	44.9 ± 5.1	0.0325	18.6 ± 11.1	6.4 ± 2.5	0.1412
	F	113.5 ± 19.6	102.0 ± 17.5	0.0260	41.8 ± 6.0	38.9 ± 3.6	0.1803	14.4 ± 4.0	6.8 ± 3.8	0.0495
	Avg. M–F	132.8 ± 28.4	121.6 ± 29.0	0.0004	45.2 ± 5.9	41.9 ± 5.2	0.0195	16.5 ± 7.8	6.6 ± 2.8	0.0440

**Table 3:** Number of patients, biological sex, age, AST (U/L), ALT (U/L), γ GT (U/L), glycaemia (U/L), triglycerides (mg/dL), total cholesterol (mg/dL), LDL (mg/dL), HDL (mg/dL), and ketone bodies in each group of patients.

	Code	Sex	Age	AST (U/L)		ALT (U/L)		γ GT (U/L)		Glycemia (U/L)		Triglycerides (mg/dL)		Total Cholesterol (mg/dL)		LDL (mg/dL)		HDL (mg%)		Ketone Bodies
				T0	T1	T0	T1	T0	T1	T0	T1	T0	T1	T0	T1	T0	T1	T0	T1	
F0–F1	Responder																			
	1	F	46	14	11	12	13	17	12	92	70	66	76	189	160	130	114	49	41	+
	2	M	36	19	40	27	68	25	23	86	75	47	80	124	117	73	59	45	42	+
	3	F	18	20	17	19	19	9	9	93	80	53	40	157	166	80	86	66	72	−
	4	M	62	16	17	22	16	13	10	104	96	112	56	177	121	124	79	35	34	−
	5	F	23	25	21	51	37	37	18	89	70	194	123	212	182	157	130	31	27	+
	6	F	32	17	21	24	31	18	14	86	89	87	84	193	158	138	111	51	45	+
	7	F	39	12	16	16	12	15	14	100	89	101	73	175	199	108	127	61	50	−
	8	M	29	53	35	110	94	40	33	83	78	130	111	256	201	182	141	48	38	+
	9	F	49	34	26	48	35	30	19	113	109	102	47	219	196	147	140	52	47	+
	10	F	51	16	15	20	16	22	19	101	98	91	88	54	43	120	126	54	43	−
	11	F	53	15	18	21	22	29	14	115	91	76	69	182	164	132	109	58	47	+
	12	F	51	18	20	12	9	30	19	89	84	89	72	204	146	127	90	64	51	+
	13	F	19	12	14	14	13	20	13	87	86	108	117	177	186	105	119	50	44	−
	14	M	25	20	22	23	31	29	27	87	83	90	92	154	147	97	94	40	37	+
	15	M	39	25	16	41	24	31	25	79	95	133	185	204	202	148	149	37	41	−
	16	F	43	18	14	21	17	51	37	95	93	128	77	212	209	160	158	36	39	−
	17	F	23	19	22	37	35	19	15	95	90	72	118	176	193	130	137	47	39	+
	18	M	44	32	33	22	20	15	11	87	89	83	68	158	189	83	112	65	70	−
	19	F	37	19	15	25	16	55	16	90	90	62	55	193	196	142	139	59	45	+
	20	F	21	16	15	16	20	13	12	94	91	115	84	150	120	69	68	76	42	+
	21	F	54	18	17	19	16	14	12	95	94	117	85	242	183	153	118	65	54	+
	22	F	53	14	21	18	43	12	14	82	95	141	78	280	239	199	186	47	53	−
	23	F	50	17	19	25	23	22	23	88	90	56	36	196	165	135	109	50	49	−
	24	M	37	22	20	38	21	38	10	105	95	67	108	167	170	85	108	59	44	+
	25	F	53	17	18	18	14	14	7	92	96	237	94	190	128	115	72	39	44	+
	26	F	50	12	11	12	9	10	6	86	83	71	48	204	163	105	94	94	61	+
	27	F	42	12	14	16	21	17	16	95	97	126	73	246	200	174	145	55	49	−
	28	F	32	18	19	19	19	14	9	78	76	90	52	211	122	153	36	48	36	+
	29	M	26	31	26	56	33	32	17	75	71	191	122	214	161	144	110	41	38	−
	30	F	57	18	22	15	18	16	11	82	77	83	54	210	183	134	112	67	54	−
	31	F	38	17	22	15	19	12	12	92	77	68	65	179	142	99	73	75	60	+
	32	F	61	14	18	18	14	17	17	89	80	142	92	193	141	125	94	42	37	−
	33	M	61	18	15	19	18	23	15	115	100	169	114	219	208	151	137	35	48	−
	34	F	38	17	13	19	15	14	18	92	81	64	52	232	156	149	97	70	49	+
	Average	9M + 25F		19.6 ± 8.0	19.5 ± 6.4	26.1 ± 18.6	24.4 ± 16.9	22.7 ± 11.4	16.1 ± 6.9	92.1 ± 9.8	87.0 ± 9.4	104.7 ± 44.0	82.0 ± 30.4	192.6 ± 40.4	166.4 ± 37.0	128.6 ± 31.0	111.1 ± 30.9	53.3 ± 14.0	46.2 ± 9.6	
	*p* value				0.9734		0.6983		0.0049		0.327		0.0158		0.0068		0.0232		0.0174	
	Non-Responder																			
	35	F	33	12	12	12	13	18	11	93	85	81	74	162	153	106	106	43	41	+
	36	F	33	13	16	12	21	12	10	98	97	61	41	163	122	108	68	59	51	+
	37	F	21	19	11	32	16	13	13	90	91	96	52	205	184	104	100	82	74	+
	38	F	47	21	16	27	17	18	13	87	86	76	106	166	158	103	107	49	47	−
	39	F	60	15	17	13	18	26	21	103	101	66	65	174	178	114	101	59	64	+
	40	F	43	17	14	18	15	15	11	100	106	80	53	191	166	129	97	72	64	−
	41	F	32	23	21	26	17	21	11	77	79	95	48	150	139	91	81	60	51	+
	42	M	25	56	20	106	42	63	32	86	83	120	97	180	175	126	125	35	35	+
	43	M	48	19	18	26	30	55	24	90	86	85	103	226	206	155	151	57	47	−
	44	M	46	18	13	30	19	45	19	97	94	261	111	341	236	237	173	47	44	−
	45	M	38	27	23	47	24	32	16	95	95	330	216	156	151	88	90	28	31	+
	46	F	55	35	21	49	18	61	22	96	92	96	63	234	178	151	117	69	63	+
	47	F	36	13	18	12	15	18	12	91	99	90	103	190	176	130	127	47	41	−
	48	F	62	20	17	20	11	25	19	87	85	101	69	210	215	130	143	68	59	+
	49	F	21	35	35	19	11	16	12	105	92	66	62	170	172	106	105	61	53	+
	50	F	54	20	24	24	21	15	11	93	82	81	77	250	229	160	135	78	81	+
	Average	4M + 12F		22.7 ± 11.2	18.5 ± 5.8	29.6 ± 23.3	19.3 ± 7.7	28.3 ± 17.6	16.1 ± 6.2	93.0 ± 7.1	90.8 ± 7.6	111.6 ± 74.4	83.8 ± 41.9	198.0 ± 48.2	177.4 ± 31.4	127.4 ± 31.0	114.1 ± 27.2	57.1 ± 15.0	52.9 ± 13.8	
	*p* value				0.1950		0.1030		0.0137		0.4048		0.2024		0.1617		0.2526		0.4109	
	Responder																			
F2	51	M	44	28	25	39	34	48	21	109	86	137	83	194	156	140	101	44	39	+
	52	M	50	47	26	105	42	23	21	105	104	98	126	203	193	124	126	52	51	−
	53	F	34	22	18	32	20	22	13	85	75	86	71	181	123	134	69	45	42	+
	54	M	21	29	29	55	36	21	13	90	84	93	69	164	153	102	94	43	45	+
	55	F	53	30	25	33	30	16	10	100	95	83	47	164	134	88	71	59	55	+
	56	M	44	50	28	90	35	76	24	94	110	174	121	209	172	151	109	40	37	−
	57	M	32	34	35	59	79	58	53	95	94	191	132	226	191	159	152	32	30	−
	58	F	40	17	14	15	13	9	11	73	82	43	65	144	141	84	90	55	52	−
	59	M	49	16	22	27	43	20	19	98	100	111	92	204	195	163	138	60	44	+
	60	M	52	59	34	105	47	49	15	106	90	102	86	208	162	145	104	47	46	+
	61	M	33	54	70	97	159	40	35	115	90	271	176	198	186	158	115	40	35	+
	Average	8M + 3F		35.1 ± 15.1	29.6 ± 14.7	59.7 ± 33.8	48.9 ± 40.2	34.7 ± 20.9	21.4 ± 12.7	97.3 ± 11.8	91.8 ± 10.2	126.3 ± 63.8	97.1 ± 37.7	190.5 ± 24.4	164.2 ± 25.3	131.6 ± 28.6	106.3 ± 25.8	47.0 ± 8.7	43.3 ± 7.7	
	*p* value				0.4010		0.5024		0.0851		0.2610		0.2063		0.0222		0.0409		0.2987	
	Non-Responder																			
	62	F	56	18	17	20	14	13	10	97	94	150	145	233	201	159	133	44	39	−
	63	F	50	24	23	34	24	18	12	91	93	102	108	217	272	164	190	53	42	−
	64	F	57	15	14	20	18	18	15	113	95	101	63	231	196	148	115	63	67	+
	65	M	64	16	22	18	23	16	10	89	93	68	50	221	204	144	119	74	76	+
	66	F	26	29	24	68	47	27	13	96	96	80	53	193	174	129	127	48	38	−
	67	F	37	12	14	12	10	7	8	85	76	64	57	172	128	102	77	66	50	+
	68	F	20	33	11	35	12	23	9	100	91	101	101	134	129	80	78	58	44	+
	69	F	33	22	14	30	14	13	8	92	87	79	70	220	139	151	87	61	42	+
	70	F	25	12	15	12	22	20	20	97	83	266	174	222	205	130	140	35	30	+
	71	F	55	20	18	11	11	13	9	84	71	91	63	162	143	97	78	59	53	+
	72	M	42	19	14	24	18	18	14	106	98	60	85	192	175	140	125	47	43	+
	Average	2M + 9F		20.0 ± 6.7	16.9 ± 4.3	25.8 ± 16.4	19.4 ± 10.4	16.9 ± 5.4	11.6 ± 3.7	95.5 ± 8.7	88.8 ± 8.7	105.6 ± 58.8	88.1 ± 40.5	199.7 ± 32.2	178.7 ± 43.3	131.3 ± 27.2	115.4 ± 34.3	55.3 ± 11.2	47.6 ± 13.4	
	*p* value				0.2116		0.2827		0.0150		0.0892		0.4247		0.2115		0.2427		0.1615	
	Responder																			
F3–F4	73	F	57	19	24	27	28	60	21	92	90	75	70	193	180	107	89	95	79	−
	74	M	44	29	29	34	53	22	19	102	98	257	176	196	174	128	102	42	42	−
	75	M	54	33	28	41	28	25	12	116	89	378	78	220	130	119	77	33	41	+
	76	M	33	35	21	57	40	82	32	109	88	181	136	207	185	147	142	39	29	+
	77	F	20	21	18	19	17	10	7	86	88	84	158	155	151	91	69	62	47	+
	78	F	33	26	34	51	59	53	38	99	82	113	78	167	124	117	85	34	30	+
	Average	3M + 3F		27.2 ± 6.4	25.7 ± 5.8	38.2 ± 14.4	37.5 ± 16.2	42.0 ± 27.4	21.5 ± 11.7	100.7 ± 10.9	89.2 ± 5.2	181.3 ± 118.2	116.0 ± 46.4	189.7 ± 24.4	157.3 ± 26.3	118.2 ± 18.9	94.0 ± 26.0	50.8 ± 24.1	44.7 ± 18.3	
	*p* value				0.6800		0.9414		0.1232		0.0422		0.2362		0.0519		0.0958		0.6278	
	Average Tot	26M + 52F		23.0 ± 10.9	20.8 ± 8.6	32.4 ± 24.2	27.1 ± 21.8	26.2 ± 16.6	16.6 ± 8.3	94.1 ± 9.7	88.9 ± 8.8	115.2 ± 64.9	88.0 ± 37.0	194.2 ± 37.6	169.4 ± 37.6	128.4 ± 30.1	110.3 ± 29.5	53.3 ± 14.2	47.2 ± 11.8	
	*p* value				0.1628		0.1501		<0.0001		0.0005		0.0016		<0.0001		0.0002		0.0044	

## Data Availability

The data are available on request from the authors.
